# 2536: The effect of skeletal muscle lipoprotein lipase overexpression on energy expenditure during weight loss maintenance and weight regain

**DOI:** 10.1017/cts.2017.54

**Published:** 2018-05-10

**Authors:** David M. Presby, Rebecca M. Foright, Julie A. Houck, Ginger C. Johnson, L. Allyson Checkley, Vanessa D. Sherk, Michael C. Rudolph, Robera M. Oljira, Matthew R. Jackman, Paul S. MacLean

**Affiliations:** University of Colorado Anschutz Medical Campus, Aurora, CO, USA

## Abstract

OBJECTIVES/SPECIFIC AIMS: Obesity is a rapidly growing epidemic and long-term interventions aimed to reduce body weight are largely unsuccessful due to an increased drive to eat and a reduced metabolic rate established during weight loss. Previously, our lab demonstrated that exercise has beneficial effects on weight loss maintenance by increasing total energy expenditure above and beyond the cost of an exercise bout and reducing the drive to eat when allowed to eat ad libitum (relapse). We hypothesized that exercise’s ability to counter these obesogenic-impetuses are mediated via improvements in skeletal muscle oxidative capacity, and tested this using a mouse model with augmented oxidative capacity in skeletal muscle. METHODS/STUDY POPULATION: We recapitulated the exercise-induced improvements in oxidative capacity using FVB mice that overexpress lipoprotein lipase in skeletal muscle (mLPL). mLPL and wild type (WT) mice were put through a weight-loss-weight-regain paradigm consisting of a high fat diet challenge for 13 weeks, with a subsequent 1-week calorie-restricted medium fat diet to induce a ~15% weight loss. This newly established weight was maintained for 2 weeks and followed with a 24-hour relapse. Metabolic phenotype was characterized by indirect calorimetry during each phase. At the conclusion of the relapse day, mice were sacrificed and tissues were harvested for molecular analysis. RESULTS/ANTICIPATED RESULTS: During weight loss maintenance, mLPL mice had a higher metabolic rate (*p*=0.0256) that was predominantly evident in the dark cycle (*p*=0.0015). Furthermore, this increased metabolic rate was not due to differences in activity (*p*=0.2877) or resting metabolic rate (*p*=0.4881). During relapse, mLPL mice ingested less calories and were protected from rapid weight regain (*p*=0.0235), despite WT mice exhibiting higher metabolic rates during the light cycle (*p*=0.0421). DISCUSSION/SIGNIFICANCE OF IMPACT: These results highlight the importance of muscular oxidative capacity in preventing a depression in total energy expenditure during weight loss maintenance, and in curbing overfeeding and weight regain during a relapse. Moreover, our data suggest that the thermic effect of food is responsible for the differences in metabolic rate, because no differences were found in activity or resting metabolic rate. Additional studies are warranted to determine the molecular mechanisms driving the ability of oxidative capacity to assist with weight loss maintenance.

